# Transcriptomic analyses of labial glands and gut tissue from two wax moths, *Achroia grisella* and *Galleria mellonella*

**DOI:** 10.1093/g3journal/jkag109

**Published:** 2026-04-25

**Authors:** Reginald Young, Khandaker Asif Ahmed, Leon Court, Rahul Rane, Tom Walsh, Gunjan Pandey

**Affiliations:** CSIRO Environment, Acton, Australian Capital Territory 2601, Australia; CSIRO Australian Animal Health Laboratory, Geelong, Victoria 3220, Australia; CSIRO Environment, Acton, Australian Capital Territory 2601, Australia; CSIRO Health and Biosecurity, Parkville, Victoria 3052, Australia; CSIRO Environment, Acton, Australian Capital Territory 2601, Australia; CSIRO Environment, Acton, Australian Capital Territory 2601, Australia

**Keywords:** wax moth, bioremediation, plastic, genome assembly, transcriptomics, enzymes

## Abstract

Plastics are highly stable materials with widespread applications, but their resistance to degradation poses a significant environmental challenge, often resulting in accumulation in landfills or pollution in the form of microplastics. Biodegradation using insect larvae has recently emerged as a promising strategy to address this issue, though the molecular basis of plastic degradation in these organisms remains poorly understood due to limited genomic resources. In this study, we present a complete genome of the lesser wax moth, *Achroia grisella*, and tissue-specific RNA-Seq data of both the lesser and the greater wax moth, *Galleria mellonella*, 2 species known to consume various plastics. Our analyses reveal several highly expressed secretory enzymes in the gut and labial tissues. Orthologous comparisons of differentially expressed genes also identified 5 enzymes (3 hexamerins and 2 monooxygenases) from the lesser wax moth that have been shown or are predicted to have plastic-degrading potential in the greater wax moth. We also identified candidate proteins showing sequence similarity to bacterial enzymes involved in polyethylene and polystyrene degradation, suggesting potential pathways for further functional investigation. Together, these genomic and transcriptomic resources provide a foundation for understanding plastic degradation in wax moths and highlight candidate genes for future functional validation.

## Introduction

The end-of-life management for plastics remains a significant environmental challenge, primarily due to the inefficiency of recycling methods and inherent durability of these synthetic polymers (Hopewell et al. 2009). A novel approach to plastic recycling that has shown potential involves the use of insects that feed on a number of plastics, including polyethylene, polystyrene, and polypropylene (Boctor et al. 2024). Notably, the larvae of several lepidopteran and coleopteran insect species, such as *Tenebrio molitor*, *Plodia interpunctella, Zophobos morio*, and *Galleria mellonella* (the greater wax moth), have been shown to exhibit plastic-feeding behavior (Yang et al. 2014; Bombelli et al. 2017; Brandon et al. 2018; Sun et al. 2022). Another species of interest is *Achroia grisella*, a close phylogenetic relative of *G. mellonella* within the same tribe (Galleriini), which similarly exhibits polyethylene-feeding capabilities (Kundungal et al. 2019), likely due to their natural diet of beeswax. However, in contrast to *G. mellonella*, research on *A. grisella* is limited by a lack of high-quality genomic and transcriptomic resources, which are crucial for understanding, optimizing, or transferring its plastic-digesting capabilities. These resources can also be used to demarcate the plastic-degrading contributions from the host animal and its microbiome, which is a topic of contention in the bioremediation field (Wang et al. 2022; He and Liu 2024).

Several proteins of interest have been previously identified in *G. mellonella* larvae to possess plastic-degrading capabilities. These enzymes, known as hexamerins, are large, secreted protein complexes containing multiple putative metallocentres. Hexamerins possess both oxygenase/dehydrogenase activity and have the ability to both append oxygen to long-chain hydrocarbons and cleave carbon–carbon (C–C) bonds. There is experimental evidence demonstrating the use of hexamerins to catalyze the transformation of polyethylene into shorter-chain hydrocarbons with terminal ketone groups (Sanluis-Verdes et al. 2022; Spínola-Amilibia et al. 2023). Given the remarkable activity of these enzymes on otherwise recalcitrant polyethylene, complete characterization of these proteins is required, and identification of similar enzymes or coenzymes involved in this process is essential to understand how nature can break down polyolefin plastics. Both moth species may also be capable of digesting plastics that are more amenable to depolymerization, such as polyethylene terephthalate and polyurethane; therefore, enzymes such as esterases and peptidases may also be of interest.

In this study, we generated a high-quality reference genome assembly for *A. grisella* and performed tissue-specific transcriptomic profiling of both *A. grisella* and *G. mellonella* larvae. Similar transcriptomic studies have been carried out on *G. mellonella*; however, to our knowledge, comparable transcriptomics resources remain limited for *A. grisella* and for larval labial glands in *G. mellonella* (Kong et al. 2019; LeMoine et al. 2020). By analyzing gene expression patterns and predicting secreted proteins across both species, we identified enzymatic candidates likely to participate in digestion. Furthermore, we characterized novel and unannotated transcripts, assigned functional annotations where possible, and examined sequence similarity to known bacterial plastic-degrading proteins to propose putative polyethylene and polystyrene degradation pathways. This work establishes a genomic and transcriptomic framework for investigating the biochemical basis of plastic degradation in wax moths.

## Materials and methods

### Insect rearing

The *G. mellonella* and *A. grisella* colonies used in this study were generously provided by Angela Ruffell, Anna Marcora, and Gene Wijffels from the CSIRO Ecosciences Precinct, Dutton Park, Queensland, Australia. The larvae of both wax moth species were maintained on a diet composed of retail-grade yeast, honey, glycerol, and baby cereal (Farex®). The environmental conditions were standardized with a 14:10 h light–dark cycle, a constant temperature of 26 °C, and 50% relative humidity.

### DNA extraction

For short-read sequencing, genomic DNA was extracted from a single lesser wax moth larva using the Qiagen DNEasy Blood and Tissue kit (Cat. #: 69504; Qiagen, Germany), animal tissue protocol. The DNA was eluted in 100 µL of 10 mM Tris-Cl, pH 8.5 (Qiagen Buffer elution buffer [EB], Cat. #: 19086) and subjected to Illumina short-read sequencing.

For long-read sequencing, high-molecular-weight genomic DNA was extracted from a single lesser wax moth larva using the Qiagen Genomic Tip 20/G kit (Qiagen, Cat. #: 10223) and the Qiagen Buffer Set (Qiagen, Cat. #: 19060). The “User-developed protocol” for “mosquitoes and other insects” supplied by Qiagen was used, except that the EB was used to dissolve the purified genomic DNA instead of Tris-Ethylenediaminetetraacetic acid (EDTA) buffer.

### *A. grisella* genome sequencing

The genomic DNA sample for short-read sequencing was submitted to Azenta Life Sciences (Suzhou facility, China) for Illumina PCR-based library construction. The library was then sequenced at the facility to approximately 60-fold genome coverage on an Illumina NovaSeq 6000 sequencer, S4 flow cell lane (2 × 150 bp PE).

The high-molecular-weight genomic DNA sample for long-read sequencing was submitted to the Biomolecular Resource Facility (BRF) at the John Curtin School of Medical Research, Australian National University. At the facility, the Circulomics SRE XS protocol (Cat. #: SS-100-121-01; PacBio, USA) was used to deplete shorter DNA fragments (<5 kb) from the sample. The size-selected genomic DNA was then prepared for sequencing on the Oxford Nanopore Technology (ONT) platform using the ONT native barcoding workflow, SQK-LSK109 kit (Oxford Nanopore, UK), and sequenced on an ONT PromethION Sequencer, FLO-PRO002 flow cells.

### *A. grisella* genome assembly and annotation

Assembly of the *A. grisella* genome was carried out similarly to the assembly of the *G. mellonella* genome (Young et al. 2024). Adapter sequences of the short-read sequencing data were removed from the resulting reads using TrimGalore v0.6.6 (Krueger 2015). The long-read sequences were trimmed using Porechop v0.2.4 (Wick et al. 2017). The long reads were assembled using SMARTdenovo v1 (Vaser et al. 2017) with Smith–Waterman free dot matrix alignment (-e dmo), kmer size of 16 (-k 16), and minimum read length of 1 kb (-J 1000). The resulting genome assembly was then subjected to 3 rounds of polishing using long reads and 3 rounds using short reads with Racon v1.4.22 (Vaser et al. 2017) under default settings. This was followed by 6 rounds of short-read-based polishing using Polca MaSURCA v4.0.7 (Zimin et al. 2013) using default settings. The resulting genome sequence was sequentially decontaminated using the NCBI Foreign Contamination Screen (FCS) tool (fcs-adaptor and fcs-gx) to obtain the final contig assembly (Astashyn et al. 2024). This assembly completeness was assessed using BUSCO v5.2.2 (Simão et al. 2015 ) against the Lepidoptera (lepidotptera_odb10) and Insecta (insecta_odb10) lineages’ gene sets. Repetitive sequence analysis was carried out using RepeatModeler2 (Flynn et al. 2020) and RepeatMasker v4.1.2 (Smit et al. 2015).

This Whole Genome Shotgun project has been deposited at DDBJ/ENA/GenBank under the accession JAULSB000000000. The genome was annotated using the NCBI Eukaryotic Genome Annotation Pipeline (EGAP; Software version: 10.1). For the gene prediction, 271 transcript sequences from EST were passed to Gnomon. Additionally, an aggregate of 349,844,374 *A. grisella* RNA-Seq reads originating from the whole organism was also used for gene prediction with STAR as part of the EGAP annotation pipeline. The full annotation details are available through NCBI Datasets (GCF_030625045.1-RS_2023_08).

### RNA extraction

**Fig. 1.**
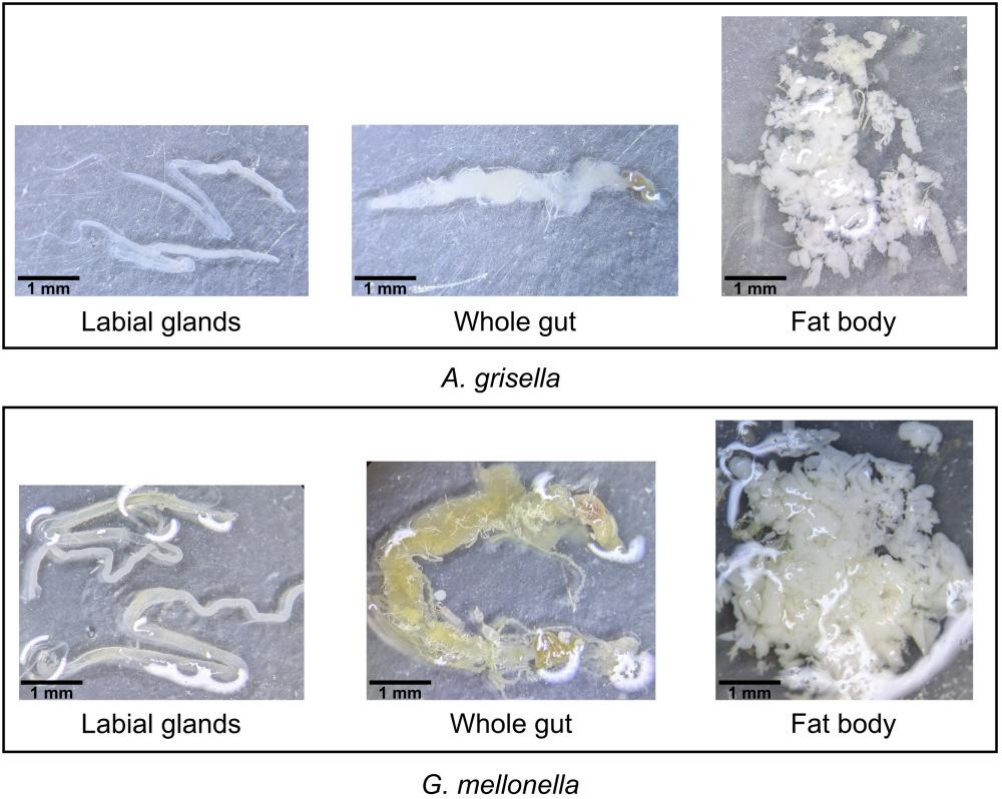
Microscopic images of labial glands, whole gut and fat body extracted from *A. grisella* (top) and *G. mellonella* (bottom) larvae.

For RNA-Seq analysis, larvae at approximately fourth to fifth instar (75 to 120 mg) were dissected to collect labial glands, whole gut, and fat body samples (Fig. 1). Larvae were picked from colonies and immediately euthanized by placing them in 50-mL centrifuge tubes on dry ice for approximately 10 min before dissection. Tissue dissections and RNA extractions were performed on the same day. A total of 15 larvae from each species (*A. grisella* and *G. mellonella*) were dissected, with tissues pooled to create 3 biological replicates for each sample type. Each biological replicate for labial glands and gut consisted of tissues pooled from 5 larvae, while fat body replicates were pooled from 3 larvae. The dissected tissues were immediately frozen on dry ice and stored in separate 1.5-mL Eppendorf tubes. Total RNA was extracted using the Zymo Quick-RNA Tissue/Insect Microprep purification kit (Cat. #: R2030; Zymo, USA), following the manufacturer's instructions.

### Transcriptomics

The set of 18 RNA extractions was stabilized and prepared using GeneWiz RNA stabilization tubes (Cat. #: GTR5025-GW; Azenta Life Sciences, China), as per the manufacturer's protocol, and subsequently provided to Azenta Life Sciences for commercial RNA sequencing. RNA-Seq libraries were prepared using poly(A)-mRNA selection and strand-specific protocols with unique dual indexing. Sequencing was performed on an Illumina NovaSeq 6000 platform using S4 flow cell lanes, generating paired-end reads (2 × 150 bp).

Quality assessment of the raw RNA-Seq reads was performed using FastQC v0.12.1 and MultiQC v1.19 (Andrew 2010; Ewels et al. 2016). Adaptor sequences and low-quality bases were trimmed using TrimGalore v0.6.6 (Krueger 2015), followed by a second round of quality control with FastQC and MultiQC. The cleaned reads were aligned to the corresponding wax moth genome assemblies (*Galleria mellonella*: GCF_026898425.1; *Achroia grisella*: GCF_030625045.1) using HISAT2 v2.2.1 (Kim et al. 2019). The resulting SAM files were sorted and converted to BAM format using SAMtools v1.18.0 (Danecek et al. 2021).

Transcript assembly and quantification were conducted using StringTie v2.2.1 (Pertea et al. 2015) in the default novel gene discovery mode, allowing for the identification of both known (annotated) and novel transcripts. Further, IsoformSwitchAnalyzeR v.1.17.04 R package was used to resolve the few ambiguities raised by StringTie (Vitting-Seerup and Sandelin 2019). This dual approach allowed for comprehensive transcriptome profiling, capturing both known and novel transcripts.

Transcript assembly completeness was assessed using BUSCO v5.4.7 against the Lepidoptera (lepidotptera_odb10) and Insecta (insecta_odb10) lineage gene sets (Simão et al. 2015). Percent alignment of transcripts to the reference genomes was assessed using Bowtie2 v2.5.4 (Langmead and Salzberg 2012).

### Differential gene expression analysis

Differential expression analyses were performed using the DESeq2 v1.42.0 R Package with gene count matrices generated by further processing the StringTie output files using the IsoformSwitchAnalyzeR v.1.17.04 R package (Pertea et al. 2015; Vitting-Seerup and Sandelin 2019). For each species, differential expression was evaluated using 3 independent pairwise comparisons: (i) labial gland versus fat body, (ii) whole gut versus fat body, and (iii) whole gut versus labial gland. For each comparison, the count matrix was subset to include only the 2 relevant tissues.

Genes with very low read counts (sum of <CPM) across all samples were filtered prior to analysis to reduce noise. A negative binomial generalized linear model was fitted with tissue as the explanatory variable (design = ∼tissue). Count data were normalized for library size differences using the default DESeq2 size-factor method. Statistical significance was assessed using the Wald test, and *P*-values were adjusted for multiple testing using the Benjamini–Hochberg false discovery rate (FDR) procedure. Genes with an absolute log2 fold change (L2FC) >2 and FDR <0.01 were considered significantly differentially expressed.

Volcano plots of the differential gene expression analyses were generated using the EnhancedVolcano v1.28.2 R package (Blighe et al. 2018). To visualize global gene expression patterns, normalized gene counts for each species were $log_{2}(x+1)$ transformed and heatmaps were generated using the pheatmap v.1.0.13 R package (Kolde 2025).

### Assignment of secretory signal peptides and gene ontology

For downstream SP prediction and functional analysis, the longest protein per gene was extracted, which involves extracting the longest protein ID per gene using “ncbi_cds2nucfile.py” python script within Orthonome pipeline (Rane et al. 2017) and extraction of corresponding protein sequences by Ids using grep function of Seqkit (v2.5.1) (Shen et al. 2016).

Secretory proteins for each genome were predicted using SignalP v6 (Teufel et al. 2022), with the organism parameter set as Eukaryota. Functional annotation was performed by EggNOG-mapper v2.1.12 with search criteria of a 0.001 minimum *e*-value, 60% identity, and minimum 50% query and subject coverage (Huerta-Cepas et al. 2019). The taxonomic scope was set to Diptera. Semantic similar Redundant Gene Ontology (GO) terms were removed, and offspring GO terms were summarized to higher level-4 Biological Process (BP), Molecular Function (MF), and Cellular Component (CC) terms with the GO.db v.3.14.0 and GSEBase v.1.56 R packages (Wu et al. 2021). The enrichGO function of the ClusterProfiler v.4.2.2 R package was used to identify the proteins with different GO terms. For Kyoto Encyclopedia of Genes and Genomes (KEGG) pathway and enzyme activity analysis, the corresponding terms were extracted from the EggNOG-mapper output, and similar over-representative analysis was performed. Names and activities of proteins of interest were extracted from the enzyme-database website (https://www.enzyme-database.org/class.php) (McDonald et al. 2009).

### Ortholog identification

Protein orthologs were identified by retrieving the longest isoforms of each gene in the .gff annotation files for each wax moth species, as well as novel transcripts identified through StringTie via ORFfinder (NCBI). These files were input into OrthoFinder v2.5.4 (Emms and Kelly 2019) using the default parameters. Key differentially expressed genes (DEGs) were then searched in the OrthoFinder output to identify orthologs between *A. grisella* and *G. mellonella*.

### BLAST searches

BLAST+ (v2.16.0) was used to identify homologous proteins using the Swiss-Prot database and manually compiled bacterial alkane and polystyrene-degrading proteins. The latter was compiled from NCBI search results of relevant proteins originating from bacteria in the same genus as reported by Ji et al. (2013) and Wang et al. (2024).

## Results

### *Achroia grisella* genome assembly and annotation

Whole-wax moth larvae were used to extract genomic DNA and subsequent sequencing. A total of 49 Gb of sequencing data was generated from whole-genome sequencing utilizing 150 base-pair paired-end Illumina short-reads (33 Gb) and Nanopore long-reads (16 Gb). The assembled genome spans 442.9 Mb in total length, with an N50 value of 3.1 Mb distributed among 525 contigs. This assembly had a consensus quality score (QV) of >40, as determined by Polca (MaSURCA v4.0.7) (Zimin et al. 2013). When analyzed for Benchmarked Universal Single-Copy Orthologs (BUSCOs), the assembled genome was 99.3% and 98.6% complete with Lepidoptera (lepidoptera_odb10) and Insecta gene sets (insecta_odb10), respectively. Table 1 contains a summary of the genome and annotation statistics for *A. grisella* as compared to the previously sequenced *G. mellonella* (Young et al. 2024). A visual representation of the genome assembly statistics is shown in Supplementary Fig. 1 as a snail plot. Analysis of repetitive sequences found in the *A. grisella* genome was also carried out; the details of which can be found in Supplementary Table 1.

**Table 1.** Summary of genome assembly and annotations statistics for the *A. grisella* and *G. mellonella* genome assemblies (Young et al. 2024).

	*Achroia grisella* (GCF_030625045.1)	*Galleria mellonella* (GCF_026898425.1)
Assembly level	Contig	Contig
Sequencing technology	Illumina HiSeq, PromethION	Illumina HiSeq, PromethION
Genome size (Mb)	442.9	471.9
Largest fragment (Mb)	10.07	15.3
Average fragment size (Mb)	0.84	2.13
N count	5	23
Gaps (b)	5	23
Genome coverage (x)	100	42
Number of contigs	525	220
Contig N50 (Mb)	3.1	6.4
Contig L50	43	25
GC content (%)	33.5	33.5
Number of Organelles (Mitochondrion)	1	1
Mitochondrion size (kb)	15.36	15.32
Number of genes	15,140	15,138
protein coding	13,873	13,604
Non-coding	312	848
tRNA	328	337
rRNA	74	77
pseudo	437	199
other	116	73
Number of transcripts	18,927	23,142
Number of proteins	18,245	23,155
GENOME: Lepidoptera_odb10 BUSCO (*n* = 5286)		
Overall score (S%+D%)	98.7	99.3
Complete and single-copy (S%)	96.1	98.1
Complete and duplicated (D%)	2.6	1.2
Fragmented (F%)	0.3	0.1
Missing (M%)	1.0	0.6
GENOME: Insecta_odb10 BUSCO (*n* = 1367)		
Overall score (S%+D%)	98.6	98.8
Complete and single-copy (S%)	96.3	98.0
Complete and duplicated (D%)	2.3	0.8
Fragmented (F%)	0.6	0.3
Missing (M%)	0.8	0.9

Annotation performed by NCBI's Eukaryotic Genome Annotation Pipeline (EGAP) identified a total of 15,140 genes and pseudogenes, including 13,873 protein-coding and 830 non-coding elements. A total of 18,232 transcripts were annotated across these genes, 2,354 of which exhibited up to 50 multiple transcript variants, although the majority only had 24 isoforms at most. The comprehensive genome annotation report can be accessed on the NCBI website (RefSeq ID: GCF_030625045.1).

### Transcriptomic profiling of labial gland and gut in wax moth larvae

Labial glands, gut tissue, and fat body were dissected and obtained from euthanized wax moth larvae and subsequently subjected to total RNA extraction followed by RNA-Seq (Fig. 1). A total of 29.7 and 29.1 Gb of 150 bp paired-end short-read, tissue-specific transcriptomic data were generated for *A. grisella* and *G. mellonella*, respectively (Supplementary Table 2). The quality of both RNA-seq datasets was assessed by aligning the reads to their respective reference genomes. For *A. grisella*, approximately 95% of reads from the fat body and gut tissue were successfully mapped, while 90% of reads from the labial gland tissue were mapped (Supplementary Table 3). Similarly, in *G. mellonella*, approximately 95% of reads from the fat body and gut samples, and 93% of reads from the labial glands were mapped to the reference genome that we assembled previously (Young et al. 2024) (Supplementary Table 3).

Completeness of the reference-guided transcriptomes was assessed using BUSCO analyses with Lepidoptera and insecta lineages for the transcriptomes of both species (Simão et al. 2015). The BUSCO analysis for *A. grisella* revealed a high level of completeness at 99.5% and 100% when assessed using the Lepidoptera and insecta BUSCO lineages, respectively, while *G. mellonella* was 99.3% and 99.8% complete using the same datasets (Supplementary Table 4).

The characterization of both reference-guided and novel tissue-specific transcripts, the latter identified through the RNA-Seq assembling software StringTie, provided further insights into species differences (Pertea et al. 2015). For the *A. grisella* reference-guided transcriptome, 16,903 to 18,727 total transcripts and 5,266 to 6,509 transcripts with multiple isoforms were identified in each tissue sample (Supplementary Table 5), reflecting the diversity of expressed genes. For the *G. mellonella* reference-guided transcriptome, 19,383 to 22,967 total transcripts and 9,456 to 12,412 transcripts with multiple isoforms were identified among the sequenced tissues (Supplementary Table 5).

The RNA-Seq data were used to identify novel transcripts not represented in the current NCBI EGAP reference annotation. These transcripts can represent entirely new genes, previously uncharacterized or alternatively spliced isoforms of known genes, or regulatory non-coding RNAs. Using StringTie we identified 4,177 and 2,218 novel transcripts in *A. grisella* and *G. mellonella*, respectively.

SignalP analyses were performed on both reference-guided and novel transcripts to identify secreted proteins that are more likely to act exogenously in the saliva or gut lumen, facilitating the digestion of ingested material, including both food and xenobiotics (Teufel et al. 2022). The total number of genes encoding proteins with secretory signal peptides (SPs) is similar between the 2 species, with 3,288 (2,316 reference and 972 novel transcripts) identified in *A. grisella* and 3,249 (2,586 reference and 663 novel transcripts) in *G. mellonella*.

### Analysis of tissue-specific transcripts

**Fig. 2.**
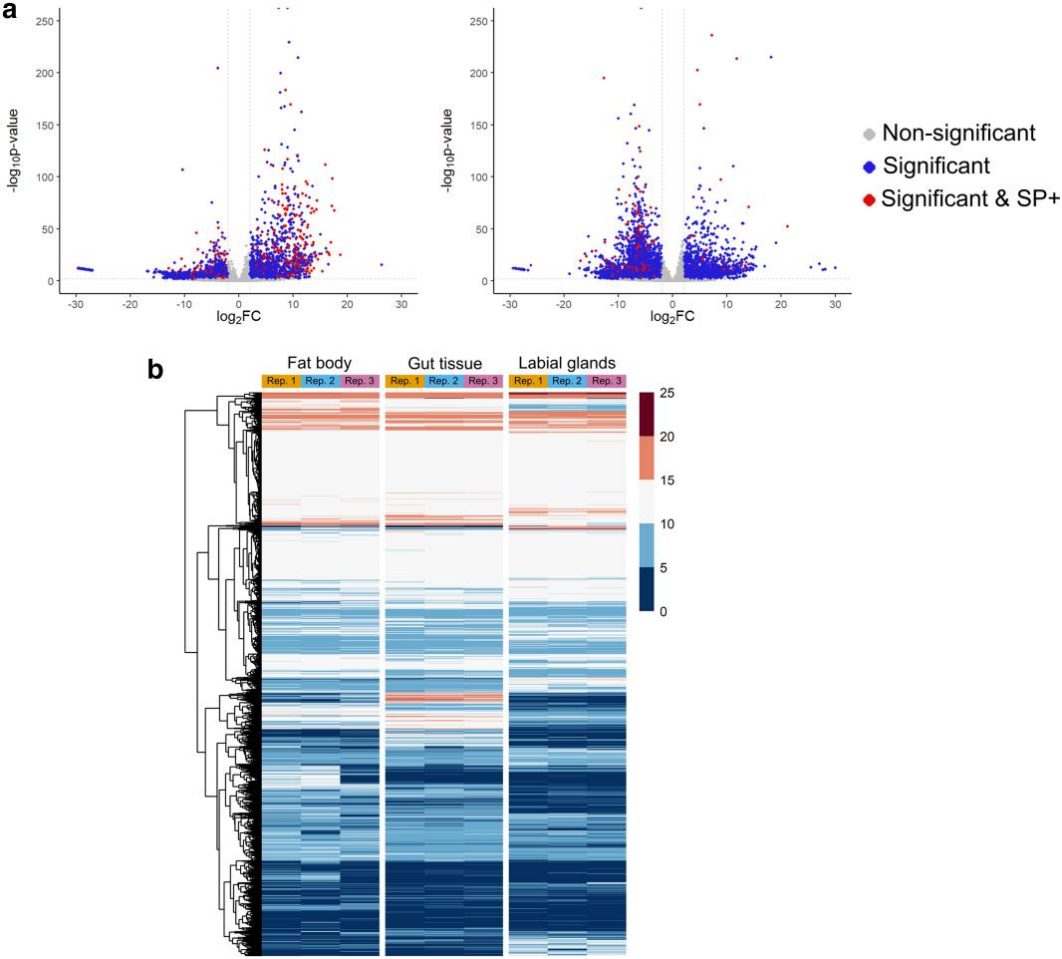
a) Volcano plots (gut tissue (left) and labial glands (right) determined relative to fat body) visualizing DEGs with and without SPs and (b) heatmap visualizing normalized DEGs from all 3 tissues for *A. grisella*. For the volcano plots, only DEGs that satisfied both L2FC (> |2|) and *P*-value (< 0.01) were considered significant. The bar on the right of the heatmap represents the $log_{2}(x+1)$ read counts per gene across different samples and replicates. *A. grisella* gut: 3,008 significant DEGs (1,357 positive and 1,651 negative L2FC); *A. grisella* labial glands: 4,721 significant DEGs (1,291 positive and 3,430 negative L2FC).

Differential gene expression analysis in the labial glands and gut tissue relative to fat body, coupled with the identification of secretory SPs, was subsequently conducted on the tissue-specific transcriptomic data. DEGs encoding secreted proteins are more likely to produce enzymes that act exogenously in the saliva or gut lumen, facilitating the digestion of ingested material, including food and xenobiotic substances such as plastics (Amezian et al. 2021). Using a threshold of L2FC >2 and *P* < 0.01, a total of 3,008 protein-coding DEGs were identified in the gut tissue of *A. grisella*, comprised of 1,357 DEGs with a positive L2FC and 1,651 with a negative L2FC (Fig. 2; Table 2). In the labial glands, 4,721 protein-coding DEGs were identified, comprising 1,291 with a positive L2FC and 3,430 with a negative L2FC. Among these, 539 and 1,124 novel transcript DEGs identified via StringTie were included in the total numbers for the gut and labial glands, respectively. Of the DEGs upregulated in the gut, 381 out of 1,357 with a positive L2FC contained a secretory SP, while a much smaller proportion, 84 out of 1,291, of the positively expressed labial gland DEGs contained a secretory SP. This aligns with the primary role of the gut in digestion and catabolism compared to the saliva of insects (Holtof et al. 2019; Jing et al. 2020).

**Table 2.** Summary of differentially expressed genes (DEGs) found in digestive tissues of *A. grisella* and *G. mellonella*, with fat body as the reference.

					Protein-coding DEGs											
					RefSeq annotated								Novel			
		Total DEGs			Positive L2FC				Negative L2FC				Positive L2FC		Negative L2FC	
	Tissue	Non-coding	Coding		Characterized		Uncharacterized		Characterized		Uncharacterized					
			Total	SP+	Total	SP+	Total	SP+	Total	SP+	Total	SP+	Total	SP+	Total	SP+
*Achroia* ***grisella***	Gut	175	3008	786	768	235	286	114	880	244	535	130	303	32	236	31
	Labial glands	425	4721	509	465	37	225	21	1970	241	937	127	601	26	523	57
*Galleria* ***mellonella***	Gut	186	2585	912	719	285	251	95	876	321	361	179	177	11	201	21
	Labial glands	313	3700	1073	486	110	179	54	1804	584	728	296	215	5	288	24

Numerous enzymes (and other proteins annotated as “enzyme-like” by the EGAP) are significantly upregulated in both tissues of *A. grisella*. In the gut, a wide variety of enzymes including 43 proteases/peptidases (33 with SP), 16 hydrolases (6 with SP), 22 esterases (13 with SP), 31 lipases (28 with SP), 24 reductases (none with SP), 16 dehydrogenases (none with SP), and 35 transferases (9 with SP) were positively, differentially expressed (Supplementary Table 6). There are comparatively fewer enzymes in the labial glands, containing mostly proteins such as fibroin and transporters but enzymes such as 14 proteases/peptidases (4 with SP), 7 hydrolases (2 with SP), 13 lipases (4 with SP), and 25 transferases (2 with SP) were present among the DEGs with a positive L2FC values greater than 2 and *P*-value of less than 0.01.

**Fig. 3.**
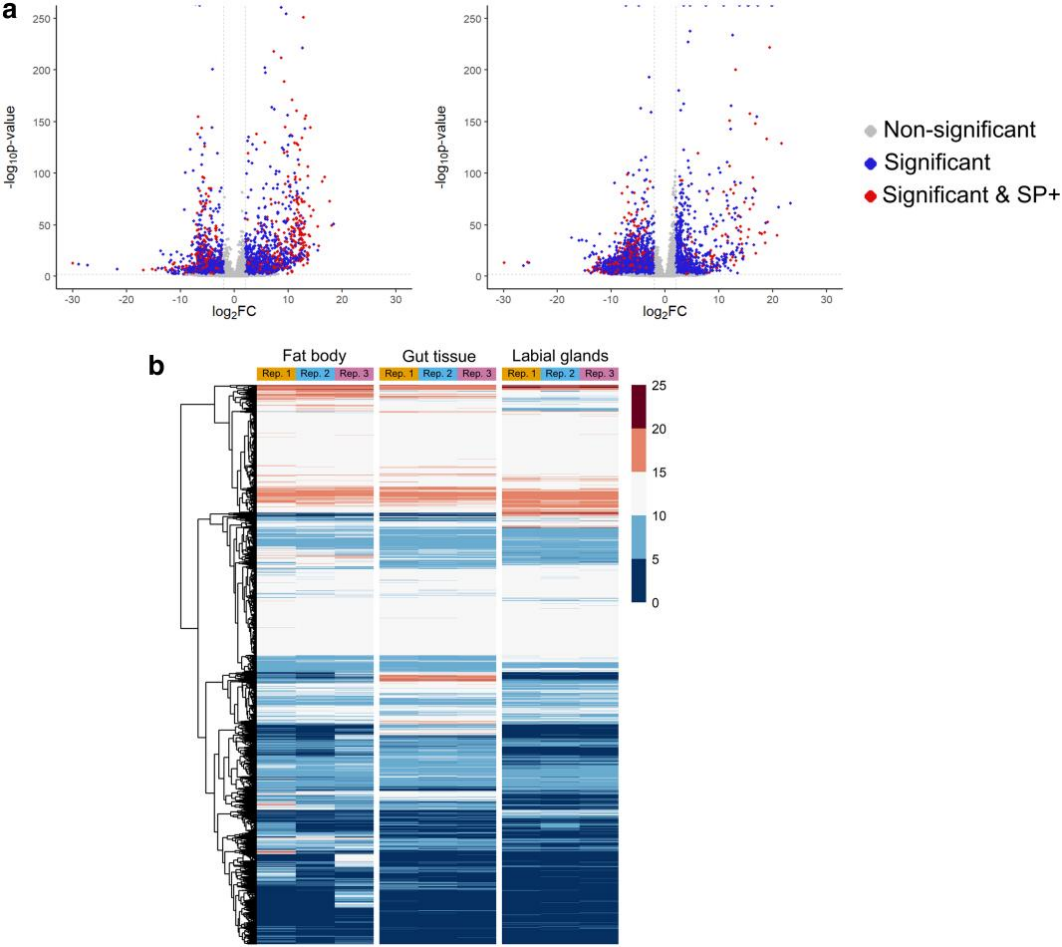
a) Volcano plots (gut tissue (left) and labial glands (right) determined relative to fat body) visualizing DEGs with and without SPs and (b) heatmap visualizing normalized DEGs from all 3 tissues for *G. mellonella*. For the volcano plots, only DEGs that satisfied both L2FC (> |2|) and *P*-value (< 0.01) were considered significant. The bar on the right of the heatmap represents the $log_{2}(x+1)$ read counts per gene across different samples and replicates. *G. mellonella* gut: 2,585 significant DEGs (1,147 positive and 1,438 negative L2FC); *G. mellonella* labial glands: 3,700 significant DEGs (880 positive and 2,820 negative L2FC).

Differential gene expression and secretion SP analysis was similarly carried out on the transcripts from gut tissue and labial glands of *G. mellonella* larvae. A total of 2,585 protein-coding DEGs (1,147 positive and 1,438 negative L2FC) in the greater wax moth gut, and 3,700 protein-coding DEGs (880 positive and 2,820 negative L2FC) in the labial glands were identified using identical threshold conditions as *A. grisella* (L2FC > |2|, *P* < 0.01) (Fig. 3; Table 2). Compared to *A. grisella*, *G. mellonella* exhibited a smaller proportion of novel transcript DEGs relative to the total DEGs, with 378 identified in the gut and 503 in the labial glands. The secretion profile of the *G. mellonella* DEGs was similar compared to *A. grisella* with more proteins bearing secretory SPs in gut tissue compared with labial glands, although a larger proportion of labial DEGs were secreted in this organism; 391 out of 1,147 in the gut and 164 out of 880 in labial glands. Each tissue also contains a largely distinct set of DEGs, particularly among the DEGs that are highly expressed within their respective tissue.

Similar to the *A. grisella* gene expression profile, *G. mellonella* gut tissue expressed a wider range of catabolic enzymes than the labial glands. Among the DEGs with positive L2FC values in the greater wax moth gut are enzymes such as 47 proteases/peptidases (42 with SP), 6 hydrolase (2 with SP), 19 esterases (16 with SP), 22 lipases (all with SP), 22 reductases (1 with SP), 11 dehydrogenases (none with SP), and 40 transferases (12 with SP), which make up a similar enzyme profile to the lesser wax moth gut (Supplementary Table 6). In the labial glands, fibroin and transporters are again highly expressed alongside enzymes including 11 proteases/peptidases (6 with SP), 5 hydrolases (none with SP), 9 lipases (6 with SP), and 32 transferases (6 with SP). Interestingly, in *G. mellonella* labial glands, 2 proteins annotated as “luciferin 4-monooxygenase-like” appear among the positive DEGs, and while an ortholog of this protein is also upregulated in *A. grisella* gut, the lesser wax moth labial glands did not produce any upregulated monooxygenase enzymes.

Differential expression of the gut and labial transcripts was also directly analyzed relative to each other, as use of fat body as a reference may mask genes involved in fatty acid and lipophilic compound degradation (LeMoine et al. 2020). In *A. grisella*, this analysis identified an additional 673 DEGs, of which 403 are gut-associated (80 with SP) and 270 are labial gland-associated (42 with SP). In *A. grisella* gut, an additional 7 proteases/peptidases (4 with SP), 2 hydrolases (1 with SP), 2 esterases (1 with SP), 14 reductases (1 with SP), 10 dehydrogenases (2 with SP), and 17 transferases (1 with SP) were identified. In the labial glands, a further 2 proteases/peptidases (both with SP), 1 secreted hydrolase, 2 lipases (1 with SP), and 5 transferases (none with SP) were identified. Similarly, for *G. mellonella*, this analysis identified an additional 942 DEGs, of which 655 are gut-associated (197 secreted) and 287 are labial gland-associated (79 secreted). In the gut, an additional 9 proteases/peptidases (4 with SP), 4 hydrolase (1 with SP), 4 esterases (1 with SP), 7 reductases (2 with SP), 10 dehydrogenases (1 with SP), and 16 transferases (3 with SP) were identified while in labial glands, a further secreted hydrolase, 2 lipases (both with SP), and 3 transferases (none with SP) were identified.

Several cytochrome P450 enzymes were also differentially expressed in both wax moth species, particularly in the gut tissue. Fifteen and 14 cytochrome P450 enzymes and corresponding essential CYP redox partners were identified in the gut tissue of *A. grisella* and *G. mellonella*, respectively, while 4 and 5 were detected in the labial glands. The cytochrome P450 enzymes shared between *A. grisella* and *G. mellonella* across gut and labial gland tissues include CYP4C1, CYP4C3, CYP6B2, CYP6B5, CYP6B6, CYP6B7, CYP9E2, and CYP12A2. However, distinct enzymes were identified in each species and tissue. In *A. grisella*, unique cytochrome P450 enzymes expressed in the gut include CYP4D8, CYP6A2, and CYP306A1, while only CYP18A1 was specific to the labial glands. In *G. mellonella*, CYP9F2 was the only unique cytochrome P450 enzyme which was upregulated in the labial glands.

Non-coding DEGs and DEGs annotated as uncharacterized were also identified in the differential expression analysis. Approximately a third of all non-novel DEGs in both tissues of *A. grisella* and more than a quarter in *G. mellonella* tissues were annotated by EGAP as uncharacterized, representing a significant number of potential genes that may contribute to the growing list of candidate enzymes of interest. Non-coding RNAs likely involved in processes such as gene regulation and differentiation were also identified (Xiao et al. 2024), with noticeably more found in the labial DEGs of both species relative to the gut tissue (Table 2).

A list of significant DEGs accompanied by relevant analyses is provided in Supplementary File 2. A complete list of the DEGs for both species is provided in Supplementary File 3, which includes L2FCs, standard L2FC error estimates, raw *P*-values, and Benjamini–Hochberg adjusted *P*-values.

### Uncharacterized and novel transcript annotation

As many uncharacterized (annotated as such by EGAP) and novel proteins (identified via StringTie; see Materials and Methods) were identified among the DEGs, an effort was made to determine the function of these proteins that lacked proper annotations. A BLASTp search against the Swiss–Prot database was carried out using the default parameters of BLAST + to determine the functions of these uncharacterized proteins (Camacho et al. 2009; Consortium 2025). This returned hits for approximately 11% (320 out of 2,807) of the uncharacterized DEGs in *A. grisella* and approximately 12% (226 out of 1,860) in *G. mellonella* at an *e*-value cutoff of 1 × 10^−20^. Several hits of note were obtained from this BLAST search, of which 22, 5, 16, and 7 were both positively differentially expressed and contained a secretory SP in *A. grisella* gut, labial glands, *G. mellonella* gut, and labial glands, respectively (Supplementary Tables 7 and 8). The L2FC of these proteins ranged from positive 2 to 17 and included carboxypeptidases, lipases, proteases, superoxide dismutases, and transferases. Among the uncharacterized proteins that remain unannotated, 308 of 2,487 and 414 of 1,634 have SPs in *A. grisella* and *G. mellonella*, respectively.

### Gene ontology and biological pathway assignment of transcripts

GO terms were assigned to the wax moth larvae DEGs to categorize their function (Table 3). Most of these putative enzymes are categorized under the BP GO term, ranging from the metabolism of amino acids to pheromones, and some are also included under the MF GO term, which encompasses enzymes such as monooxygenases and hydrolases. Transcripts that are positively upregulated and contain secretory peptides were limited to the gut tissue, including hydrolase and peptidase enzymes, and others involved in catabolism. Other GO terms that are not necessarily associated with enzymatic activities, such as CC terms, are also highlighted as they may include proteins of interest. Furthermore, other terms such as DEGs associated with hormone biosynthesis and neurotransmitter metabolism are highlighted as these processes often involve oxidation reactions (Sen et al. 2003; Pan et al. 2021).

**Table 3.** The number of differentially expressed genes (DEGs) identified in wax moth larvae tissue corresponding with GO terms associated with metabolism.

		*Achroia grisella*						*Galleria mellonella*					
		Gut			Labial glands			Gut			Labial glands		
	GO term	All	SP+	SP+ and upregulated	All	SP+	SP+ and upregulated	All	SP+	SP+ and upregulated	All	SP+	SP+ and upregulated
**Biological Process**	Cellular amino acid metabolic process				52			33			51		
	Cellular chemical homeostasis	27											
	Cellular modified amino acid metabolic process	15						14					
	Hormone biosynthetic process	14											
	Lipid metabolic process							60					
	Neurotransmitter metabolic process				15			11			14		
	Organic acid metabolic process				107			80			119		
	Organic hydroxy compound metabolic process							28					
	Organic substance catabolic process		40	25					44				
	Organonitrogen compound metabolic process								100	47			
	Pheromone metabolic process	7			8			6					
	Protein metabolic process									37			
	Small molecule biosynthetic process							51			63		
	Small molecule catabolic process	26			44			30			47		
**Molecular Function**	Hydrolase activity, acting on carbon-nitrogen (but not peptide) bonds							19	9		23		
	Hydrolase activity, acting on glycosyl bonds	24		14	26			26	26	15	26		
	Monooxygenase activity	10			17			12			15		
	Oxidoreductase activity, acting on paired donors, with incorporation or reduction of molecular oxygen				19			16					
	Oxidoreductase activity, acting on single donors with incorporation of molecular oxygen	6											
	Oxidoreductase activity, acting on the aldehyde or oxo group of donors				13			10			15		
	Oxidoreductase activity, acting on the CH-NH group of donors	6											
	Peptidase activity	57		23				66	51	33			
	Serine hydrolase activity	21						33	29	18	31		
**Cellular Component**	Extracellular region	51			76			64	53		68		

DEGs in a given GO term, DEGs with secretory signal peptides (SP+) and DEGs with both secretory signal peptides and positive L2FC values (upregulated) are counted separately.

GO term assignment on the *G. mellonella* DEGs also revealed several putative proteins that may be involved in plastic degradation but also highlighted differences between the 2 moth species (Table 3). The DEGs of *G. mellonella* were assigned more unique GO terms, and exclusively expressed proteins involved in lipid metabolism, protein metabolism, and metabolism of organic compounds containing hydroxyl groups and nitrogen. Similar to *A. grisella*, proteins with secretory SPs and positive upregulation were limited to the gut tissue, including those involved in the metabolism of organic compounds, hydrolase and peptidase enzymes. Contrastingly, in *A. grisella* tissue, unique GO terms such as proteins involved in catalyzing redox reactions involving CH–NH groups and maintenance of chemical homeostasis were assigned. GO terms that are shared between the species also exhibited differences in tissue localization and presence of secretory SPs.

Filtering the DEGs to include only those assigned enzymatic terms reveals hydrolases, lyases, and oxidoreductases as the 3 main classes of enzymes (Table 4). Hydrolases appear to be the most abundant class in both species, particularly those acting on ester bonds in *G. mellonella* labial glands and peptidases in the gut tissue of both species. Other enzymes of note include oxidoreductases involving the incorporation of oxygen, present in all 4 tissue groups, and oxidoreductases utilizing CH–OH groups as donors, which are present in both *G. mellonella* tissue but exclusively in *A. grisella* labial glands.

**Table 4.** The number of differentially expressed genes (DEGs) identified in wax moth larvae tissue corresponding to enzymatic GO terms for metabolism.

		*Achroia grisella*						*Galleria mellonella*					
		Gut			Labial glands			Gut			Labial glands		
Enzyme class	Enzyme function	All	SP+	SP+ and upregulated	All	SP+	SP+ and upregulated	All	SP+	SP+ and upregulated	All	SP+	SP+ and upregulated
**Hydrolase**	Acting on ester bonds										71		
	Acting on peptide bonds (peptidases)	39							27	16			
**Lyase**	Glycosylases	22						23					
**Oxidoreductase**	Acting on paired donors, with incorporation or reduction of molecular oxygen	18			28			20			21		
	Acting on single donors with incorporation of molecular oxygen (oxygenases)	7											
	Acting on the aldehyde or oxo group of donors										18		
	Acting on the CH-OH group of donors				40			33			38		

DEGs in a given GO term, DEGs with secretory signal peptides (SP+) and DEGs with both secretory signal peptides and positive L2FC values (upregulated) are counted separately.

DEGs were also assigned to biological pathways outlined in the KEGG (Table 5). DEGs of both gut and labial glands in *A. grisella* were assigned to several pathways involving the metabolism of amino acids and carbohydrates, while only the gut was involved in pathways involving hormone synthesis and oxidative phosphorylation. Interestingly, *A. grisella* labial glands exclusively contained proteins involved in carbon metabolism, which encompasses the metabolism of carbohydrates, carboxylic acids, and fatty acids. In *G. mellonella* tissue, proteins classified under lipid metabolism are more abundant compared to *A. grisella* tissue, which may be involved in the processing of structurally similar long-chain hydrocarbon xenobiotics such as polyethylene. Peroxisome-related proteins, which may be key for attaching oxygen onto carbons, were also found in both digestive tissues of *G. mellonella* (Table 5).

**Table 5.** The number of differentially expressed genes (DEGs) identified in wax moth larvae tissue corresponding with KEGG pathways of metabolic interest

		*Achroia grisella*						*G. mellonella*					
		Gut			Labial glands			Gut			Labial glands		
Higher level term	KEGG pathway	All	SP+	SP+ and upregulated	All	SP+	SP+ and upregulated	All	SP+	SP+ and upregulated	All	SP+	SP+ and upregulated
**Amino acid metabolism**	Arginine and proline metabolism	11			15						15		
	Glycine, serine and threonine metabolism	14			20			12			20		
	Phenylalanine metabolism	7						8			10		
	Tyrosine metabolism	10			15			10					
	Valine, leucine and isoleucine degradation										20		
**Carbohydrate metabolism**	Ascorbate and aldarate metabolism	9			17			16			21		
	Fructose and mannose metabolism							21			17		
	Galactose metabolism	18			20			25		11			
	Glyoxylate and dicarboxylate metabolism										15		
	Pyruvate metabolism				18								
	Starch and sucrose metabolism									7			
**Digestive system**	Carbohydrate digestion and absorption	19		7						8			
	Cholesterol metabolism							12					
	Fat digestion and absorption							12					
**Endocrine system**	Aldosterone synthesis and secretion	21											
**Energy metabolism**	Oxidative phosphorylation	36											
**Global pathways**	Biosynthesis of secondary metabolites	73			130			95			122		
	Carbon metabolism				45								
	Fatty acid metabolism				25			23			32		
	Metabolic pathways	192			280			218			261		
**Glycan biosynthesis and metabolism**	Glycosaminoglycan degradation										8		
**Lipid metabolism**	α-Linolenic acid metabolism							10			10		
	Arachidonic acid metabolism							12					
	Biosynthesis of unsaturated fatty acids				15			15					
	Fatty acid biosynthesis										9		
	Fatty acid degradation										25		
	Fatty acid elongation							10			13		
	Glycerolipid metabolism				24			25					
	Linoleic acid metabolism							6					
**Metabolism of cofactors and vitamins**	Nicotinate and nicotinamide metabolism							8					
	Retinol metabolism				11			10			15		
**Metabolism of other amino acids**	β-Alanine metabolism										13		
	Glutathione metabolism							14					
**Metabolism of terpenoids and polyketides**	Insect hormone biosynthesis	12			13			9			15		
**Transport and catabolism**	Peroxisome							25			40		
**Xenobiotics biodegradation and metabolism**	Drug metabolism—cytochrome P450				18			15			19		
	Drug metabolism—other enzymes				29			20			29		
	Metabolism of xenobiotics by cytochrome P450				22			18			24		

DEGs in a given pathway, DEGs with secretory signal peptides (SP+) and DEGs with both secretory signal peptides and positive L2FC values (upregulated) are counted separately.

### Orthologous analysis of plastic-degrading enzymes

To investigate conserved and species-specific enzymes implicated in plastic degradation, we identified orthologs between *G. mellonella* and *A. grisella*. Given their close taxonomic relations within the *Galleriini* tribe and shared natural diet of beeswax, identifying orthologs offers valuable insights into potential function conservation. Using OrthoFinder, we compared the whole proteomes of both wax moths to each other as well as against *Amyelois transitella*, a related Pyralidae moth that does not exhibit plastic-feeding behavior (Emms and Kelly 2019). *Amyelois transitella* was used as a comparative species to the wax moths as it is taxonomically related yet has a natural diet of fruit and tree nuts (Wilson et al. 2020), which is significantly distinct in chemical makeup compared to beeswax. This comparative analysis enabled differentiation between broadly conserved Pyralidae genes and wax-moth-specific genes potentially linked to beeswax and plastic consumption.

Across the dataset, over 91% of annotated proteins from both wax moths were assigned to orthogroups. Approximately 72% (16,790) of *A. grisella* genes and 76% (15,245) of *G. mellonella* genes shared orthologs with *A. transitella*, highlighting a substantial proportion of genes that are likely common among lepidopteran in the wax moths (Supplementary File 4). A summary of orthologous and non-orthologous DEG statistics for both wax moth species is provided in Supplementary Table 9.

Among the identified orthologs, several candidate enzymes implicated in plastic degradation were conserved between *G. mellonella* and *A. grisella* (Supplementary Table 10 in Supplementary File 1). Notably, 3 orthologs of the 4 previously identified plastic-degrading hexamerin enzymes from *G. mellonella* (Spínola-Amilibia et al. 2023) were detected in *A. grisella* with 70% to 80% amino acid identity. All 3 orthologs exhibited lower expression in both digestive tissues compared with fat body. Additionally, a *G. mellonella* hexamerin (XP_026756149.2), which also shows lower expression in both digestive tissues versus fat body and has not yet been described in the literature, had a corresponding *A. grisella* ortholog (XP_059052760.1) that showed no differential expression in either tissue. Other enzymes previously implicated in plastic degradation in *G. mellonella*, such as cytochrome P450 and other oxygenase enzymes (Venegas et al. 2024), also have corresponding orthologs identified in *A. grisella* (Supplementary Table 10).

Other highly differentially expressed enzymes such as peptidases and esterases as well as transferases and reductases appeared to be shared between *G. mellonella* and *A. grisella*. Many highly differentially expressed (L2FC of 2 to 18; Supplementary File 2) lipases, with distinct enzyme profiles between gut and labial glands, were detected in both species. Several lipases are upregulated in the gut but downregulated in the labial glands. However, there were also distinct labial-specific lipases for both species. Similarly, gut-specific peptidase orthologs were detected, with many highly expressed (L2FC of 2 to 14; Supplementary File 2) carboxypeptidases. Other similar hydrolases (eg aminopeptidases and a high number of collagenases) were also found. In addition, a number of transferase orthologs were also highly differentially expressed, predominantly in the gut, with L2FC values ranging from 2 to 15 in both species (Supplementary File 2).

Filtering out orthologs present in both wax moths but absent in *A. transitella* identified several wax-moth-specific enzymes. Among these, carboxypeptidases and lipases were detected in both the gut and labial glands of *G. mellonella* and *A. grisella*. Additionally, 2 copies of 15-hydroxyprostaglandin dehydrogenase (XP_026753012.1; XP_059061339.1; XP_059061618.1), luciferin 4-monooxygenase (XP_052752027.1; XP_059051626.1), and the cytochrome P450 enzyme CYP6B5 (XP_026759413.1; XP_059047416.1) were identified as unique and highly differentially expressed in labial gland of both wax moth species. Several enzymes involved in fatty acid metabolism were also specific to the wax moths (Supplementary File 2).

Among the 1,521 *A. grisella* and 894 *G. mellonella* DEGs that have no orthologs in the other wax moth species, a substantial number may be species-specific enzymes. The majority of these genes are either novel or annotated as uncharacterized, comprising 1,269 (83%) in *A. grisella* and 705 (79%) in *G. mellonella*. Among the non-orthologous DEGs with assigned annotations, proteins unique to *G. mellonella* included several carboxypeptidases, chitin deacetylases, and collagenases in the gut, as well as ecdysone oxidases and glucosyltransferases in the labial glands, with trehalose transporters present in both tissues. Similarly, non-orthologous DEGs of *A. grisella* included carboxypeptidases, chitin deacetylases, and collagenases, alongside several lipases, reductases, and dehydrogenases, and distinct labial gland-specific enzymes such as a lipase (XP_059058795.1) and a serine protease (XP_059056009.1). Species-specific cytochrome P450 enzymes were also identified in both wax moth species, with 2 gut-specific P450s detected in each (*A. grisella*: XP_059045017.1 and XP_059045042.1; *G. mellonella*: XP_052751866.1 and XP_052750885.1), and 1 (*A. grisella*: XP_059047416.1) and 3 (*G. mellonella*: XP_026749603.2, XP_052755777.1, and XP_026749508.2) uniquely upregulated in the labial glands.

**Fig. 4.**
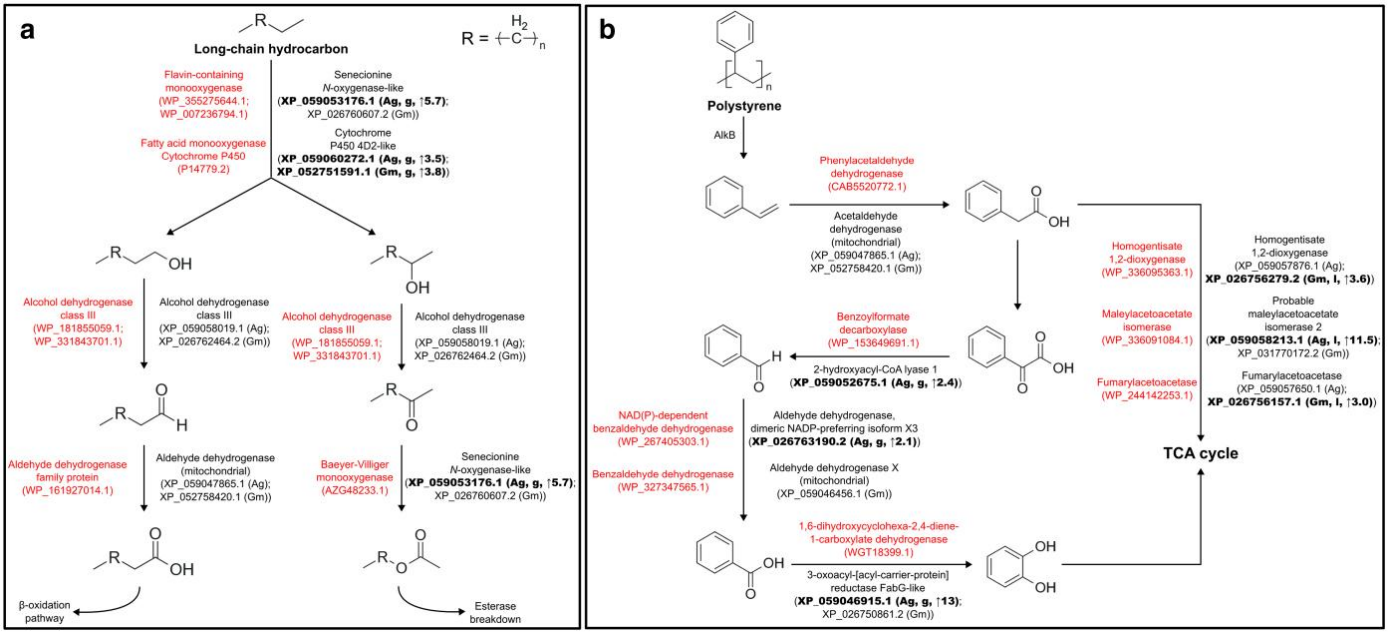
Proposed metabolic pathways of (a) long-chain hydrocarbons (e.g. polyethylene) and (b) polystyrene in bacteria, as reported by Ji et al. 2013 and Wang et al. 2024, respectively. Bacterial genes are denoted by red text and putative homologous wax moth genes are denoted by black text. For the wax moth genes, the corresponding species (either *A. grisella* as “Ag” or *G. mellonella* as “Gm”), specific tissue they are expressed in (either gut (g) or labial glands (l)) and log2-fold expression change are noted in brackets. Additionally, upregulation of wax moth genes is further highlighted with bold text. If the gene is not differentially expressed but present in the genome, no further information is included in the brackets next to the gene.

The proteomes of the reference genomes, along with novel genes from both wax moth species, were further queried using BLAST searches against known bacterial alkane- and polystyrene-degrading genes to identify homologous proteins (Supplementary File 5). Alkane-degrading enzymes catalyze the breakdown of long-chain hydrocarbons and have been implicated in bacterial degradation of plastics (Ji et al. 2013). Several gut-specific wax moth cytochrome P450 enzymes, including CYP6B2 (XP_059045017.1) and CYP4C1 (XP_059052592.1) in *A. grisella*, and CYP6B6 (XP_052751866.1) and CYP6B5 (XP_052750885.1) in *G. mellonella*, showed 25% to 30% sequence identity to P450 enzymes involved in fatty acid hydroxylation and oxygenation reactions in *Bacillus* species (Fig. 4a). Similarly, flavin-dependent monooxygenase enzymes from *Gordonia* spp., which are involved in hydrocarbon oxidation, exhibited 30% to 37% protein-level homology to an oxygenase annotated as senecionine N-oxygenase-like (XP_059053176.1) identified in the gut tissue of *A. grisella.*

Several wax moth enzymes also showed sequence homology to bacterial enzymes known to be involved in polystyrene degradation (Wang et al. 2024). In the gut tissues, wax moth dehydrogenases and reductases exhibited approximately 30% similarity to styrene-metabolizing enzymes from various *Pseudomonas* spp., including a FabG-like reductase (XP_059046913.1), 15-hydroxyprostaglandin dehydrogenase (XP_059061618.1), and D-altritol 5-dehydrogenase (XP_059057756.1) (Fig. 4b). Additionally, enzymes expressed in the labial glands of both wax moth species showed up to 50% homology to bacterial enzymes involved in downstream polystyrene metabolite transformations (Wang et al. 2024). These included maleylacetoacetate isomerase (XP_059058213.1; *A. grisella*), homogentisate 1,2-dioxygenase (XP_026756279.2; *G. mellonella*), fumarylacetoacetase (XP_026756157.1; *G. mellonella*), and a sorbitol dehydrogenase-like protein (XP_026749976.1; *G. mellonella*) (Fig. 4b).

To supplement these BLAST search results in corroborating the function of analogous wax moth proteins, conserved domain searches were carried out using these protein hits as queries. The NCBI Conserved Domain Database (CDD) identified several conserved domains among the query wax moth proteins, largely agreeing with their existing annotations (Marchler-Bauer et al. 2017). Interestingly, senecionine N-oxygenase-like (XP_059053176.1) was matched with several conserved domains, mainly those involved with the binding of redox cofactors such as flavin and NAD(P)H/NAD(P)+, indicating some sort of involvement in oxygenation reactions. Uncharacterized and novel wax moth proteins that were queried were also matched with conserved domains, providing further insight into their function. Wax moth proteins annotated as uncharacterized that shared sequence homology with alkane-degrading bacterial genes were found to have cytochrome P450 domains, while those that shared sequence homology with polystyrene-degrading bacterial genes contained several domains involved in redox reactions, particularly short-chain oxidoreductases. Similarly, novel proteins were found to be matched with cytochrome and redox enzyme domains. A CDD search with both bacterial and wax-moth-derived orthologs outlined in Fig. 4 also found that orthologs from both organisms contained domains with identical or similar annotations. The results of the conserved domain search can be accessed in Supplementary File 5.

## Discussion

This study presents a genome assembly of *A. grisella* that currently serves as the RefSeq, alongside transcriptomic analyses of digestive tissues from both *A. grisella* and *G. mellonella*. Compared to a previous draft genome assembly of *A. grisella* (Koseva et al. 2019), our assembly is more contiguous (525 scaffolds vs >3,000 scaffolds) and more complete (BUSCO completeness 99.3% vs 80.7%). While gene counts are sensitive to annotation methods, our assembly produced >15,000 predicted genes compared with >10,000 reported by Koseva and coworkers. The linkage mapping resource reported may also be useful in future work to further scaffold and orient assemblies in this species. The high-quality reference genomes for *A. grisella* and previously described *G. mellonell*a enabled a robust investigation into digestive tissue–specific gene expression and the identification of secreted catabolic enzymes with potential roles in diet and plastic degradation.

Although the salivary glands of wax moth larvae contain 2 distinct tissues, labial and mandibular glands, we opted to analyze the labial glands as they are involved in producing saliva among many lepidopteran species and are involved in a variety of functions, including digestion (Rivera-Vega et al. 2017; Mikó et al. 2019). We have identified several digestive enzymes of interest from the labial glands, suggesting that these glands may also contribute to the processing of ingested material in addition to silk production. However, the mandibular glands are purported to have specialized catabolic functions and may be more involved in producing digestive secretions in the greater wax moth (Wroniszewska 1966; Celorio-Mancera et al. 2012). As such, additional transcriptomic analyses on isolated mandibular glands from both wax moth species may reveal more specialized enzymes that could be implicated in long-chain hydrocarbon metabolism.

The fat body served effectively as the reference tissue in the DEG analysis. However, as the fat body itself is involved in various metabolic processes including lipid, carbohydrate, and amino acid metabolism, as well as the storage and utilization of biomolecules for energy (Arrese and Soulages 2010), some transcripts related to fat processing in the digestive tissues may also be highly expressed in the fat body. While the fat body was chosen as a reference due to its primary roles in biosynthesis and energy storage rather than catabolism, this functional overlap was considered in our analysis (Arrese and Soulages 2010). We have cautiously interpreted the results and also performed direct comparisons between the labial glands and gut to support our findings.

Transcriptomic data revealed clear tissue-specific gene expression patterns in both wax moth species, despite possible influences from the laboratory diet. Gut tissue exhibited the greatest diversity and abundance of DEGs encoding catabolic enzymes and secretory proteins, including peptidases, esterases, lipases, oxygenases (eg cytochrome P450s), and reductases. These findings are consistent with the primary role of the gut in insects for digestion and catabolism (Holtof et al. 2019; Jing et al. 2020). The presence of secretory SPs on many of these DEGs indicated the critical role of extracellular enzymes in digestion. In contrast, the labial glands, while containing fewer DEGs overall, may support preliminary digestion by expressing extracellular hydrolytic enzymes like lipases, peptidases, and transferases. These enzymes in both tissues may be represent candidate proteins that could potentially contribute to the biochemical transformation of polyethylene and related hydrocarbons, for example, through the addition of oxygen to the carbon chain (oxygenases) or introduction of chemically labile functional groups, such as alkenes via reduction (reductases) (Tian et al. 2017; Ghatge et al. 2020; Lu et al. 2021). Peptidase, esterase, and lipase enzymes found in these tissues could potentially act on other polymers whose chemical structures are more amenable to degradation, such as polyesters and polyamides, possibly through the cleavage of ester and amide bonds.

GO term assignment of the transcriptomic data further revealed several unique features in each species. Most of the GO terms assigned were SignalP negative DEGs, while DEGs with secretory SPs were mainly peptidases, hydrolases, and proteins involved in organic compound metabolism. In *A. grisella*, GO terms associated with hormone metabolism and xenobiotic degradation suggest adaptations to other substrates that may enhance their ability to process exogenous substrates such as hydrocarbons. In *G. mellonella*, DEGs included a higher diversity of enzymes involved in lipid and protein metabolism, which may reflect a greater metabolic flexibility.

Orthology analyses between *A. grisella* and *G. mellonella* revealed functional similarities, particularly for the known plastic-degrading enzymes. Although they consistently exhibited lower expression in the digestive tissue compared with fat body, all 4 previously characterized hexamerin polyethylene-degrading enzymes in *G. mellonella* were found to be orthologous with 3 hexamerins in *A. grisella*, sharing high sequence homology (>70%) (Spínola-Amilibia et al. 2023). Two monooxygenases orthologous to putative plastic-degrading enzymes in *G. mellonella* were also identified in *A. grisella* (Venegas et al. 2024). These findings suggest that both species may possess similar enzymatic capacity for polyethylene degradation. However, differences in DEG profiles were observed, such as the presence of luciferin monooxygenases in *G. mellonella* labial glands and unique GO terms in *A. grisella* related to hormone and pheromone metabolism, which may reflect species-specific adaptations to certain metabolic processes. Additionally, the use of the *A. transitella* genome to filter out common Pyralidae orthologs allowed for the identification of wax-moth-specific genes, which likely contain specialized proteins more suitable for biochemical reactions on plastic polymers.

Orthology analyses also identified species-specific DEGs (ie no orthologs found by OrthoFinder in the other wax moth species), which may be involved in biological processes unique to each moth. Filtering for species-specific DEGs that also contain SPs, similar protein profiles are revealed in the tissues of both species. The gut is rich with peptidase and protease enzymes such as carboxypeptidase B and Q, collagenase, and brachyurin. Species-specific enzymes capable of hydrolytically cleaving ester bonds including various lipases and esterases also appear in the gut tissue of both species. However, differences in the species specificity of DEGs are more apparent in the labial glands. Far fewer unique DEGs are found in *A. grisella* than *G. mellonella*, and the only enzymes of note that appear include an endothelial lipase (XP_059048035.1), a phosphatase (XP_059061013.1), and a serine protease (XP_059056009.1). Similar species-specific and secreted enzymes in *G. mellonella* are also found in the labial glands but others including a trypsin (XP_026756774.2) and glycosyl transferase (XP_052757042.1) are also unique to the greater wax moth. The similarity yet specificity of these enzymes to each species suggests that there may be inherent differences in the way each wax moth processes its food, with *G. mellonella* producing slightly more enzymes unique to the species.

Cytochrome P450s have also emerged as a potentially important class of enzymes for plastics bioremediation. These enzymes are known for their ability to catalyze various chemical transformations on diverse substrates, including the oxidation of dietary compounds, toxins, and xenobiotics (Nauen et al. 2022). Among those that are identified as DEGs in the wax moths, CYP4C1 and CYP4C3 are involved in fatty acid metabolism and detoxification of xenobiotics, supporting energy production and maintaining cellular homeostasis (Martins and Bitondi 2016; Tian et al. 2021). The CYP6 family, including CYP6B2, CYP6B6, and CYP6B7, is critical for metabolizing plant allelochemicals and insecticide breakdown (Munoz et al. 2014; Tian et al. 2017; Huang et al. 2023), and both CYP9E2 and CYP12A2 are also involved in insecticide and xenobiotic detoxification (Hojland et al. 2014; Kaplanoglu et al. 2024). Given their ability to oxidize natural products and insecticides with complex carbon skeletons as a result of their relaxed substrate specificity, cytochrome P450 enzymes may also have promiscuous activities on long-chain hydrocarbons, which may involve oxidation of methylene or terminal carbons in the polyethylene structure, in the wax moth as well as in other plastic-feeding insects (Behrendorff et al. 2015).

Several wax moth enzymes were identified with similar sequences to known bacterial degradation enzymes, indicating that these hits found via BLAST may also be involved in the degradation pathway of alkanes and polystyrene. Given that there are previous reports of both polyethylene and polystyrene consumption by wax moth larvae (Kundungal et al. 2019; Sanluis-Verdes et al. 2022; Spínola-Amilibia et al. 2023), it is plausible that the breakdown mechanism would mirror those that occur in microbial species. The wax moth protein hits were found to have approximately 20% to over 50% sequence similarity to the bacterial proteins. The BLAST hits include enzymes responsible for oxidation, hydroxylation, and reduction reactions, which are necessary for alkane degradation, and enzymes involved in similar reactions for the metabolism of styrene (Fig. 4). Several of the hits were also found to be differentially expressed in either gut or labial glands, particularly oxygenase, cytochrome P450, and dehydrogenase enzymes, suggesting a more involved role in these tissues. In addition, these wax moth protein hits were queried in a CDD search to further infer their function, in which matched domains of enzyme superfamilies were found to be largely in agreement with the wax moth protein annotations. A CDD search also returned identical or similar domains found in both bacterial and wax moth orthologs, outlined in Fig. 4, further corroborating that similar polystyrene and long-chain hydrocarbon-degrading functions may be possible in the wax moths. Though these BLAST and CDD searches provided some evidence of protein function, further *in vitro* investigations into these proteins are required to verify their activity as well as to identify compatible substrates and their biochemical mechanism.

We note that these similarities reflect low-to-moderate sequence identities expected for distant homologs when comparing proteins across domains (bacteria to insects) and therefore do not establish strict homology or imply that these genes are unique to wax moths. Many of the enzyme superfamilies represented among these hits (eg oxidoreductases, dehydrogenases, monooxygenases/P450s) are broadly distributed across Lepidoptera, and homologs are likely to also occur in non-plastic-feeding species. Determining whether homologs are uniquely expanded, differentially regulated, or preferentially expressed in plastic-feeding taxa would require broader comparative genomic and tissue-specific expression analyses across additional Lepidoptera under defined feeding conditions.

The accuracy of the transcriptomic analyses is heavily dependent on existing reference genomes and annotations. As reference-based alignment relies on the quality of the reference genome assembly, inconsistencies and errors in this reference would be incorporated into the aligned transcripts, resulting in incomplete downstream analyses (Lee et al. 2021). This was partially rectified using the novel gene discovery mode of StringTie, which identified thousands of novel genes in both species that were not identified by EGAP. Other assembly methods, such as *de novo* assembly of the transcripts via Trinity (Grabherr et al. 2011), may retrieve elements that were previously not found in the original genome reference. Additionally, use of other annotation pipelines or prediction tools may improve the quality of the annotated genes (Holt and Yandell 2011; Maia et al. 2022), particularly as a high number of uncharacterized protein products are present in both wax moth species. However, this may also be due to the lack of comprehensive characterization of lepidopteran proteomes, resulting in low-quality annotations for obscure genes, as reflected by most uncharacterized proteins (*A. grisella*: 2487/2807; *G. mellonella*: 1633/1860) returning no hits from a BLAST search using the SwissProt database.

As plastics are large macromolecules, they would have difficulty being internalized into cells (Jensen et al. 2003), and hence, catabolic reactions likely occur extracellularly prior to a more complete degradation intracellularly. Extracellular enzymes, including secreted peptidases, esterases, and lipases, represent candidate proteins that may contribute to the initial chemical transformation of plastics in both gut lumen and saliva. In contrast, intracellular, gut-specific enzymes that often require redox cofactors, such as reductases, dehydrogenases, and cytochrome P450s (McLean et al. 2005; Chen et al. 2014), likely act on partially digested products absorbed into gut cells. Together, these findings are consistent with a potential multistep enzymatic process involved in hydrocarbon or plastic degradation, although functional validation will be required to confirm these activities.

Although the classical protein secretory pathway via *N*-terminal SPs likely acts as the primary method of translocating proteins to the gut lumen or saliva, unconventional protein secretion may also affect which proteins can act on macromolecular substrates. Many proteins undergo secretion through unconventional pathways, including enzymes such as dehydrogenases, kinases, and proteases (Cohen et al. 2020; Maricchiolo et al. 2022), broadening the range of wax moth candidate enzymes for extracellular substrates. Additionally, unconventional protein secretion can be triggered as a stress response and therefore is relevant to plastic-fed wax moth larvae that exhibit heightened stress (Rabouille 2017). As a result, the DEGs that lacked SPs may also have relevance in the plastic degradation pathway within the wax moth larvae; however, exploration of unconventionally secreted proteins was outside the scope of this work. In parallel with the SignalP analysis, GO annotation and enzyme classification analyses (Tables 3–5) were conducted on all DEGs, irrespective of SP prediction, thereby also encompassing proteins that may be secreted through nonclassical mechanisms. The integration of SignalP, GO, and ortholog analyses together provides a comprehensive view of the degradative potential encoded within the DEG set.

Plastic degradation in wax moths is increasingly understood to be a collaborative process between the host and its microbiota. As this article focuses on the host contribution of the wax moths on polyethylene degradation, a key limitation to these studies is the contribution of the microbiome for bioremediation. Several studies have identified microbial species housed in wax moth gut that are capable of independently degrading plastics (Zhang et al. 2020; Ruiz Barrionuevo et al. 2022; Wang et al. 2022; Kehkashan et al. 2025; Ma et al. 2025). However, there are also studies that support the host as a contributor to wax catabolism (Kong et al. 2019; LeMoine et al. 2020). Future research combining host-side analyses with metagenomic and metatranscriptomic analyses will be essential to elucidate a more comprehensive understanding of the mechanism underlying plastic degradation by wax moth larvae.

These findings emphasize the need to address challenges in identifying novel polyethylene-degrading enzymes. Unlike polymers with hydrolyzable bonds, polyethylene degradation likely involves oxygen incorporation prior to bond cleavage, as suggested by the activity of hexamerins and monooxygenases (Spínola-Amilibia et al. 2023; Venegas et al. 2024). However, not all enzymes of interest may be upregulated in digestive tissue relative to fat body, and there is no single class of enzymes that is capable of all relevant oxygenation and C-C cleavage reactions on long-chain hydrocarbons. Apart from the hexamerins, the only other currently known protein capable of acting on polyethylene is a multi-copper oxidase found in the bacterium *Rhodococcus opacus* (Zampolli et al. 2023), which shares little sequence and structure similarity to the hexamerins. Hence, a comprehensive understanding of the mechanism behind biological polyethylene breakdown is crucial to aid the discovery of novel enzymes active on chemically inert plastics.

### Conclusions

This study provides a complete genome assembly of *A. grisella* and comprehensive transcriptomic analyses of digestive tissues in both *A. grisella* and *G. mellonella*. The identification of DEGs encoding catabolic enzymes, particularly secreted peptidases, esterases, and lipases, highlights the enzymatic potential of these tissues for plastic degradation. Additionally, proteins not associated with the conventional secretion pathway, including cytochrome P450s, reductases, and oxygenases, are also found to be upregulated in the digestive tissue, suggesting involvement in catabolic activities. Orthologous comparisons between the wax moth species identified candidate hexamerins and monooxygenases in *A. grisella* that likely contribute to polyethylene degradation via similar chemical mechanisms, as well as specialized enzymes unique to the wax moths by filtering common orthologs. Proteins with sequence similarities to bacterial plastic-degrading genes were also identified, giving insights into the potential mechanisms by which the wax moth larvae may biochemically degrade plastic polymers. These findings lay the groundwork for future investigations into the unique metabolic capabilities of wax moth larvae and their applications in mitigating plastic waste.

## Data Availability

The datasets discussed in this article are available in the NCBI Sequence Read Archive (SRA) repository. All genomic and transcriptomic sequencing data generated for *Achroia grisella* are available under BioProject PRJNA995347 (BioSample SAMN36468096). Transcriptomic data for *Galleria mellonella* are available under BioProject PRJNA893711 (BioSample SAMN31432028). Genome assemblies for *A. grisella* and *G. mellonella* are accessible via GenBank under accession numbers GCA_030625045.1 and GCA_026898425.1, respectively, and are associated with the above BioProjects. Scripts and code have been uploaded to GitHub and are available in the following repository: https://github.com/asifratul/Waxmoth_RNASeq. Raw and normalized (TPM) transcript counts from processed RNA-Seq data have been indexed at NCBI Gene Expression Omnibus (GEO) with accession numbers GSE322731 and GSE322730 for *A. grisella* and *G. mellonella* data, respectively. Full differential expression outputs for all DESeq2 contrasts are provided as Supplementary File 3 accompanying this manuscript. The Supplementary Files are available via GSA FigShare (https://doi.org/10.25387/g3.32071746).
